# A Multiband Antenna Stacked with Novel Metamaterial SCSRR and CSSRR for WiMAX/WLAN Applications

**DOI:** 10.3390/mi12020113

**Published:** 2021-01-22

**Authors:** Rajiv Mohan David, Mohammad Saadh AW, Tanweer Ali, Pradeep Kumar

**Affiliations:** 1 Department of Electronics and Communication, Manipal Institute of Technology, Manipal Academy of Higher Education, Manipal, Karnataka 567104, India; rajiv.md@manipal.edu; 2 School of Electronics, Vellore Institute of Technology, Vellore 632014, India; saadh.mohammad@gmail.com; 3 Discipline of Electrical, Electronic and Computer Engineering, University of KwaZulu-Natal, Durban 4041, South Africa

**Keywords:** CSSRR, metamaterial, permittivity, permeability, SCSRR

## Abstract

This paper presents an innovative method for the design of a triple band meta-mode antenna. This unique design of antenna finds application in a particular frequency band of WLAN and WiMAX. This antenna comprises of a square complimentary split ring resonator (SCSRR), a coaxial feed, and two symmetrical comb shaped split ring resonators (CSSRR). The metamaterial unit cell SCSRR independently gains control in the band range 3.15–3.25 GHz (WiMAX), whereas two symmetrical CSSRR unit cell controls the band in the ranges 3.91–4.01 GHz and 5.79–5.94 GHz (WLAN). This design methodology and the study of the suggested unit cells structure are reviewed in classical waveguide medium theory. The antenna has a miniaturized size of only 0.213$\lambda_{0}$ × 0.192$\lambda_{0}$ × 0.0271$\lambda_{0}$ (20 × 18 × 2.54 mm^3^, where $\lambda_{0}$ is the free space wavelength at 3.2 GHz). The detailed dimension analysis of the proposed antenna and its radiation efficiency are also presented in this paper. All the necessary simulations are carried out in High Frequency Structure Simulator (HFSS) 13.0 tool.

## 1. Introduction

Currently, wireless communication networks employing metamaterial technology are witnessing unprecedented development. Metamaterials are artificially structured media comprising of dielectric inclusions that can be engineered to exhibit peculiar electromagnetic properties [1,2,3,4]. These materials have been found to have significant advantages in various field of technology [5]. One such new category is the metamaterial formed by depositing metal structures on dielectric substrates. Subsequently, rather than the composition, the structure of the metamaterial decides its physical property. Left hand medium (LHM) materials investigated by Veselago in 1968 exhibit negative permittivity and permeability through magnetic, electric, and phase triplet vectors [6]. LHM materials are formed by electromagnetic wave propagation due to the parallel and opposite directionality nature of phase and group velocity to the pointing vector, and thus are unavailable in nature. Negative refractive index materials can be used for terahertz applications as demonstrated by Pendry [7].

An electromagnetic metamaterial is not available naturally, and thus is defined as artificially composed uniform structure, wherein the average cell size *p* should be much smaller than the guided wavelength λ_g_ (*p* << λ_g_). This condition is maintained to ensure that the scattering or diffraction is mainly controlled by the refractive process after the prorogation of the wave in metamaterial medium [8]. Metamaterials are mainly used to enhance the system performance and antenna parameters such as gain, bandwidth, efficiency, and return loss. Additionally, these artificially created structures also find applications in microwave and terahertz to construct devices such as integrated sensors, filters, and more [9,10]. The use of metamaterial antenna in improving the performance of the antenna is continuing to evolve rapidly in the present times [11,12,13]. Metamaterials have been found to be attractive candidates for achieving multiband operation due to their exotic properties such as negative refractive index and the possibility of tailoring permittivity and permeability. In addition to this, the near field boundary conditions of the metamaterial could be altered to achieve a compact size while retaining better radiation performance.

## 2. Literature Review

The literature reports several works on metamaterial based multiband antennas [14,15,16,17,18,19,20]. Amani et al. [14] proposed a CPW-fed composite right/left-handed resonant antenna. Although good impedance matching was achieved, lower gain of −0.5 dB and total efficiency of 70% was reported. A microstrip fed monopole antenna was designed by Huang et al. [15]; metamaterial inspired reactive loading and L shaped slots were practically used for achieving multiband operation. It was observed that the antenna dimension had large area of 40 × 45 mm^2^. Similarly, a multiband antenna of dimensions 20 × 30 mm^2^ was proposed by Yu et al. [16]; multiband operation was achieved by employing complimentary split ring resonators. However, the independent control over the achieved band was not possible. A dual antenna loaded with meandered line and square split ring resonator (SRR) was proposed in [17]. However, the antenna has a relatively larger size of 20 × 22 mm^2^ and the overall antenna structure tends to be complex. Aznabet et al. [18] proposed a large sized 30 × 22 mm^2^ metamaterial antenna just to operate at single band. The metamaterial unit cell property was not discussed and verified. A composite right/left-handed (CRLH) single-layer metamaterial antenna based on the T-junction discontinuity with a considerable size of 20 × 20 mm^2^ was presented in [19]. Although the antenna operated at triple band, a negative gain of around −0.5 dBi was observed at the lower resonance. Also, the used structure tends to be complex in nature. Similarly, three zeroth order resonator (ZOR) cells-based metamaterial antenna was proposed in [20] for dual band operations. However, the antenna has a large size of 42 × 50 mm^2^ with negative gain of −1.5 dBi at the lowest operating band.

In our investigations, a new meta-mode multiband antenna is presented. The novelty of the work lies in the proposed metamaterial unit cell which is tuned in such a way that each individual unit cell solely controls a particular resonance frequency of the antenna. This feature of the antenna makes it distinguishable with the other multiband metamaterial antennas available in literature. The other main advantages of the proposed antenna are that it provides high miniaturization (i.e., very compact size) and simple and planar configuration with high radiation efficiency and stable radiation pattern as compared to the antenna presented in Table 2. The proposed design consists of a radiator which includes two metamaterial unit cells, i.e., square complimentary split ring resonator (SCSRR) and two symmetrical comb shaped split ring resonator (CSSRR). SCSRR controls the band at 5.9 GHz, whereas CSSRR controls the band at 3.2 and 4 GHz, respectively. The metamaterial unit cell and its parameters extraction such as effective permittivity, permeability, and refractive index are studied in detail with the help of waveguide medium environment. Various studies such as split gap effect of unit cell, loading of each unit cell individually, and current distribution are also analyzed. Parametric investigations are discussed to finalize the design dimension of the antenna. The entire simulations of the antenna and metamaterial unit cell is carried out in a High frequency Structure Simulator (HFSS) v.13.0 tool.

## 3. Antenna Layout and Design

**Figure 1:**
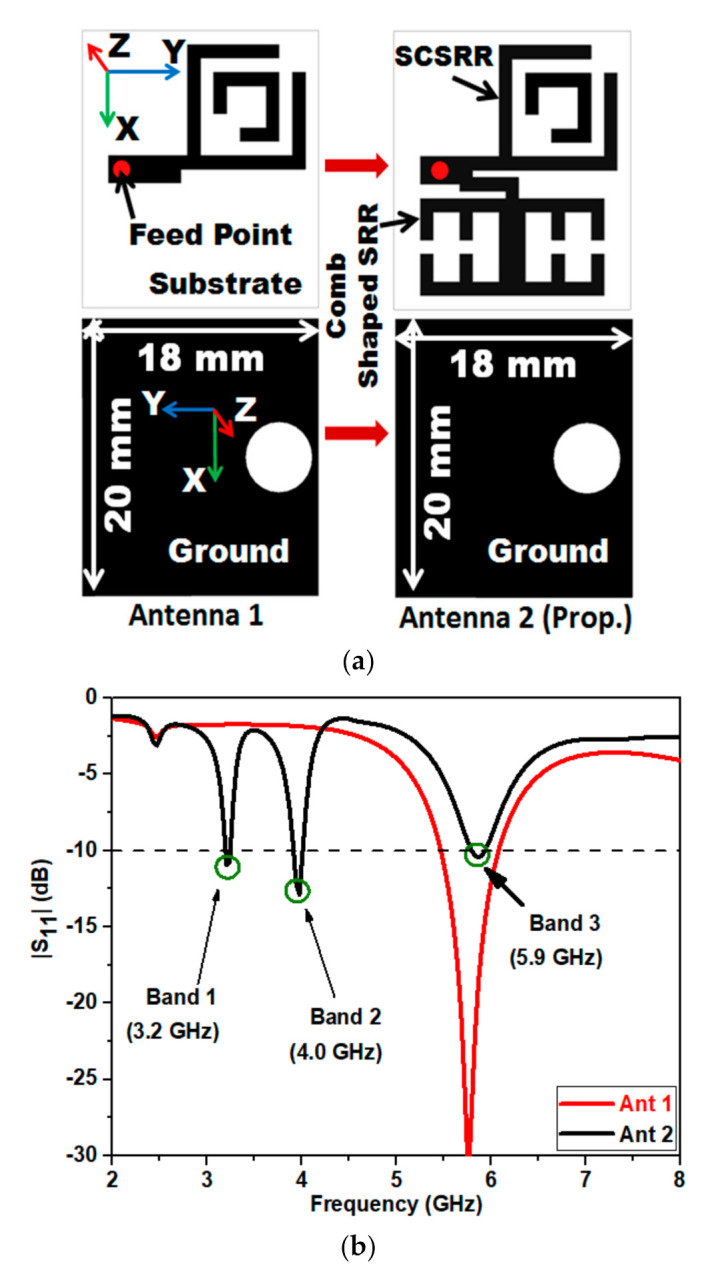
Proposed antenna design evolution steps (**a**) Antenna 1 loaded with square complimentary split ring resonator (SCSRR) and (**b**) Antenna 2 (Prop.) loaded with symmetrical comb shaped split ring resonators (CSSRR) and SCSRR.

Figure 1 describes the design evolution of the suggested metamaterial antenna consisting of a rectangular stub loaded with SCSRR as the radiating patch. Antenna 2 consists of two symmetrical CSSRR along with configuration of Antenna 1. It is observed in Figure 1a that the design is on RT Duroid 6006 substrate with ε_r_ = 6.15, height = 2.54 mm and loss tangent = 0.0019. The simulated S11 results of the antennas presented inFigure 1a is given in Figure 1b. The antenna structures shown in Figure 1a uses Finite Element Method (FEM) method with the simulation and optimization performed on HFSS) 13.0 version. Initially Antenna 1 is designed to produce resonance at 5.8 GHz (WLAN) with |S_11_| < −10 dB bandwidth ranging from 5.45–6.05 GHz (Figure 1b). However, we can see that the designed Antenna 1 has the disadvantage of single band of operation.

In order to get additional operating bands, two symmetrical CSSRR metamaterial unit cells are introduced in the design as depicted in configuration Antenna 2 of Figure 1a. It is apparent that by the inclusion of metamaterial unit cells, the solenoid current is activated along with magnetic field fluctuations resulting in magnetic response from the conductor. This resultant effect makes the antenna to further resonate at 3.2 (WiMAX) and 4 GHz (WLAN) frequency bands.

**Figure 2:**
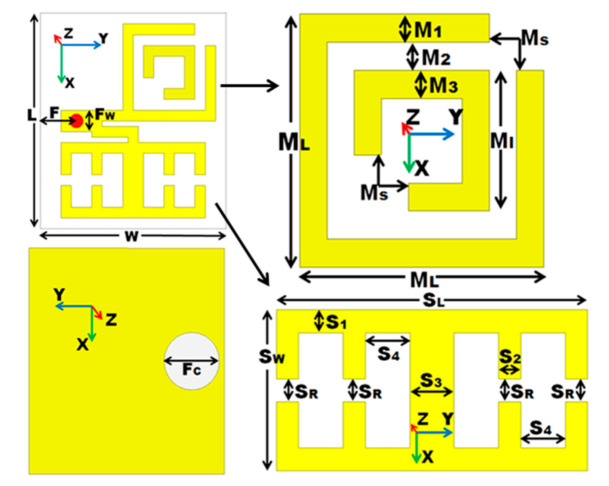
Detailed dimension layout of the proposed antenna.

Figure 2 illustrates the detailed geometry of metamaterial Antenna 2. It is evident that this proposed configuration has the advantage of a simple structure with very compact dimension along with triple useful band of operations. It is observed that this antenna is comprised of a radiating patch loaded with SCSRR and two symmetrical CSSRR which are energized by a 50Ω coaxial feed. To obtain better performance some of the key parameters are adjusted, and the optimized dimension parameters (mm) of the antenna are given in Table 1.

## 4. Parameters Extraction of Metamaterial Unit Cell Using Waveguide Medium

**Figure 3:**
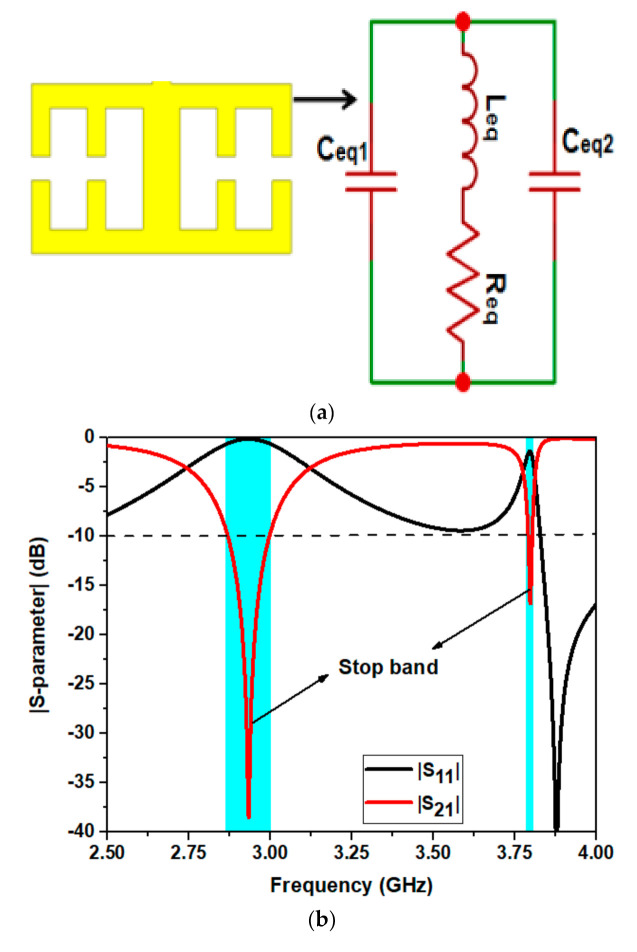

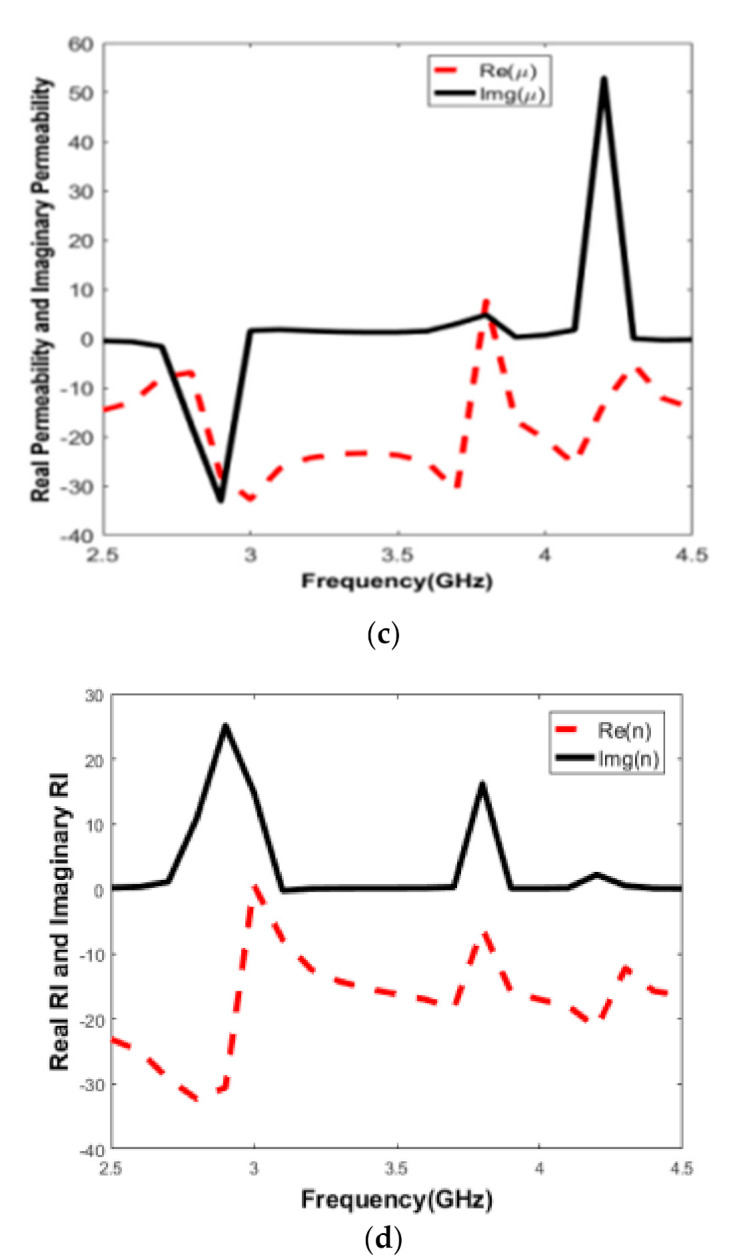
Metamaterial CSSRR analysis (**a**) Proposed CSSRR with its equivalent circuit (**b**)|S-parameter| obtained from waveguide medium (**c**) Real and imaginary effective permeability (µ*_eff_*) and (**d**) Real and imaginary effective refractive index (η*_eff_*) of the CSSRR.

Two of these symmetrical unit cell CSSRR are modeled using metallic resonators and thereby etching a bifurcated gap in the opposing sides, resulting in the desired stop band phenomenon as depicted in Figure 3a. The main advantage of utilizing CSRR metamaterial unit cell is to get multiband operation. This unit cell is formed by a symmetrical comb shaped strips (resonator) place on either side with a split gap between them. The resonators form the inductive effect and the split gaps form the capacitive effect as shown in the equivalent circuit Figure 3a. With the help of such structure this unit cell can operate at 2.9 and 3.9 GHz. The operation of this multiband behavior can be understood by studying the reflection (|S11|) and transmission (|S21|) coefficient (i.e., pass band behavior) by placing the unit cell in waveguide medium. This study is based on Nicholson–Ross–Weir (NRW) technique which was further elaborated by Smith et al. [21] to extract the material parameters utilizing waveguide medium theory. The waveguide environment is used to calculate the value of |S11|and |S21|of the proposed metamaterial unit cell CSSRR. The CSSRR is placed inside the waveguide environment where it is provided with Perfect Electric Conductor (PEC) and Perfect Magnetic Conductor (PMC) boundary conditions. The electromagnetic wave is provided at port 1 (i.e., input port) and the complex value of |S11|and |S21|are calculated using output port 2, as demonstrated in Figure 3b, wherein one can see that the stop band phenomenon is observed at 2.9 and 3.9 GHz (Since at this operating frequency |S21| is less than 10 dB and |S11| is closer to zero). Subsequently, the effective permeability (µ*_eff_*) is calculated using [2] and is demonstrated in Figure 3c.

The CSSRR resonant frequency is computed as given in (1).

(1) $$\omega_{r}=\;\frac{1}{\sqrt{L_{eq}C_{eq}}}$$

where *L_eq_*, signifies the total inductance due to the metallic ring whereas *C_eq_* denotes the total capacitance (*C_eq1_* || *C_eq2_*) of the split gap of CSSRR as represented in equivalent circuit (Figure 3a). Accordingly, the effective permeability (*µ_eff_*), impedance (Z*_eff_*) and refractive index (*η_eff_*) are computed from the S parameters using Equations (2)–(4) [21].

(2) $$\mu_{eff}=\;n_{eff}\times Z_{eff}$$

where,

(3) $$Z_{eff}=\;\sqrt{\frac{{\left(1+S_{11}\right)}^{2}-S_{21}{}^{2}}{{\left(1-S_{11}\right)}^{2}-S_{21}{}^{2}}}$$

The convention of impedance is determined by imposing the conditions Re (Z*_eff_*)) and Im (η*_ef f_*) as given in [22,23].

(4) $$n_{eff}=\frac{1}{k_{0}d}\left\{Im\left[ln\left(e^{ink_{0}d}\right)\right]+2m\pi -iRe\left[ln\left(e^{ink_{0}d}\right)\right]\right\}$$

where,

$$e^{ink_{0}d}=\frac{S_{21}}{1-S_{11}\left(\frac{z-1}{z+1}\right)}$$

$k_{0}=\frac{2\pi f}{c}$, corresponds to speed of light measured in m/s, *f* denotes the operating frequency, the branch index is denoted as m and the thickness of the substrate is given by d. The extracted *µ_eff_* and *η_eff_* is illustrated in Figure 3c,d. Henceforth, it is observed that a negative value of permeability for the real part is observed at 2.9 and 3.9 GHz, this is due to the effect of stopband phenomenon of this CSSRR. This negative value for permeability implies when the structure is used as a radiator, the antenna exhibits new frequency in terms of return loss characteristics.

Similar to this analysis, the SCSRR analysis was conducted and the negative permeability is observed at 5.9 GHz. For brevity we have not included the analysis here.

## 5. Split Gap Analysis, Individual Unit Cell (Resonator) Loading Effect, and Current Distribution

**Figure 4:**
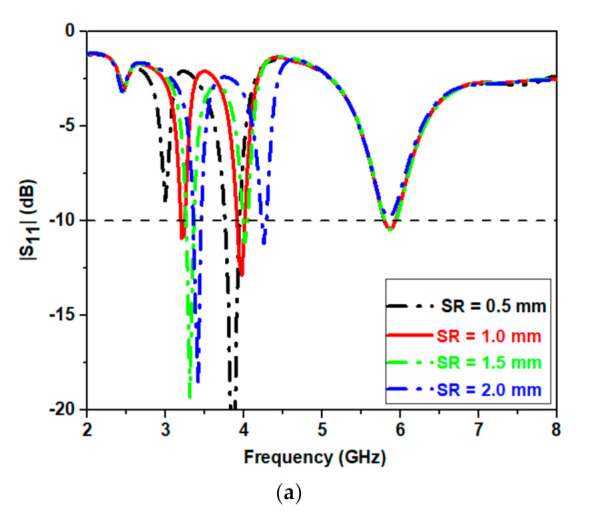

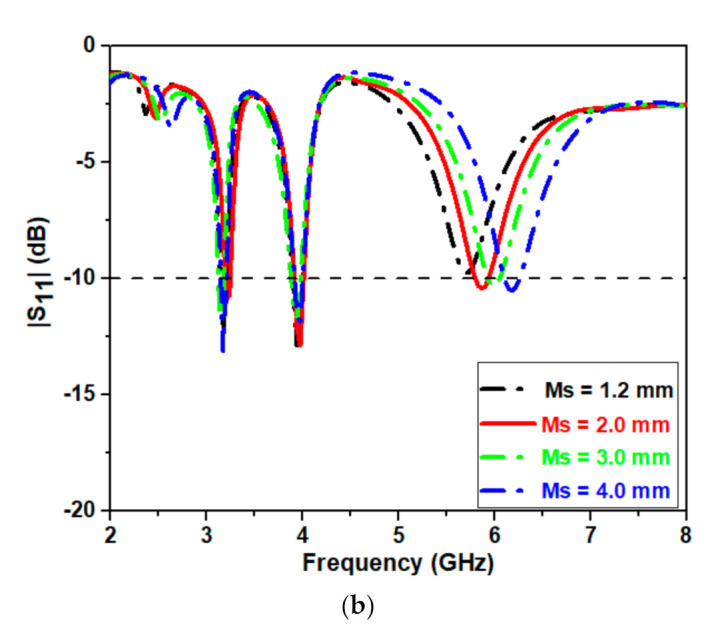
Variation of SR on (**a**) S_11_ and (**b**) M_S_.

To fix the size of both the metamaterial split gaps its parametric investigation is carried out and is explained in Figure 4. These parametric investigations provide an optimized dimension of the design of metamaterial antenna. It can be analyzed from Figure 4a that an alteration in the spilt gap width in CSSRR (S_R_) the impedance matching at all the three band is affected. At S_R_ = 0.5 mm, the antenna becomes dual band as the |S11| value at first resonance shifts above −10 dB. At S_R_ = 1.5 mm, a shift at fundamental mode is observed, decrease in the value of |S11|is observed at second mode, while there is no change observed at the third mode. For the proper operation of antenna at all the three frequency the dimension of S_R_ is fixed at 1.5 mm.

Similarly, the split gap (M_S_) analysis of the second unit cell SCSRR is carried out and is displayed in Figure 4b. It can be studied that at M_S_ = 1.2 mm, 3 mm and 4 mm, the first two modes are least affected while there is a noticeable resonance shift at third mode. For proper operation of antenna at all the three frequency the dimension of M_S_ is fixed at 2 mm. Thus, from this analysis it can be concluded that the spilt gap width in CSSRR (S_R_) and SCSRR (M_S_) perturbs the surface current distribution, which drastically affects the resonance and impedance behavior of the antenna.

**Figure 5:**
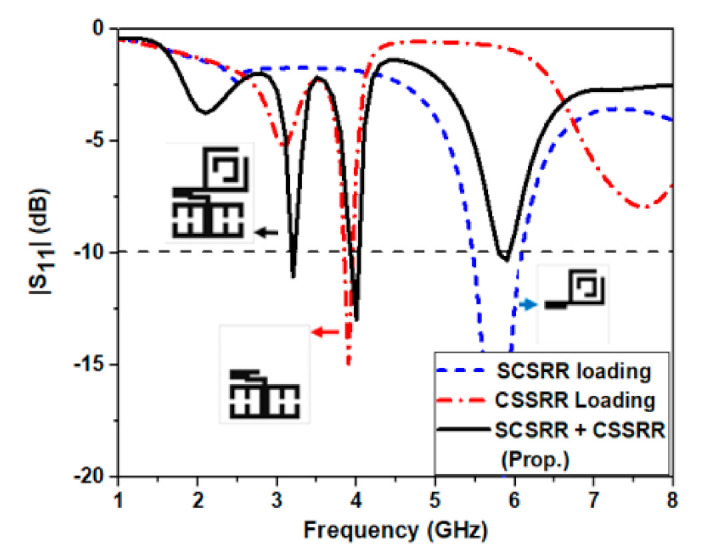
Effect of loading individual metamaterial unit cell.

To understand the loading effect of each unit cell individually and to analyze how each resonance is impacted by the other one as a parasitic element, a study is carried out and is depicted in Figure 5. From the figure it is noted that when the unit cell SCSRR is loaded individually, a narrow resonance is observed at 5.8 GHz. When only the unit cell CSSRR is loaded it shows single band operation around 4 GHz. When both the unit cells are loaded together antenna shows triple band operation (i.e., 3.2, 4 and 5.9 GHz), which is basically due to parasitic loading effect of both the unit cell simultaneously which affect the flow of surface current distribution, thereby making the antenna exhibit multiband behavior.

**Figure 6:**
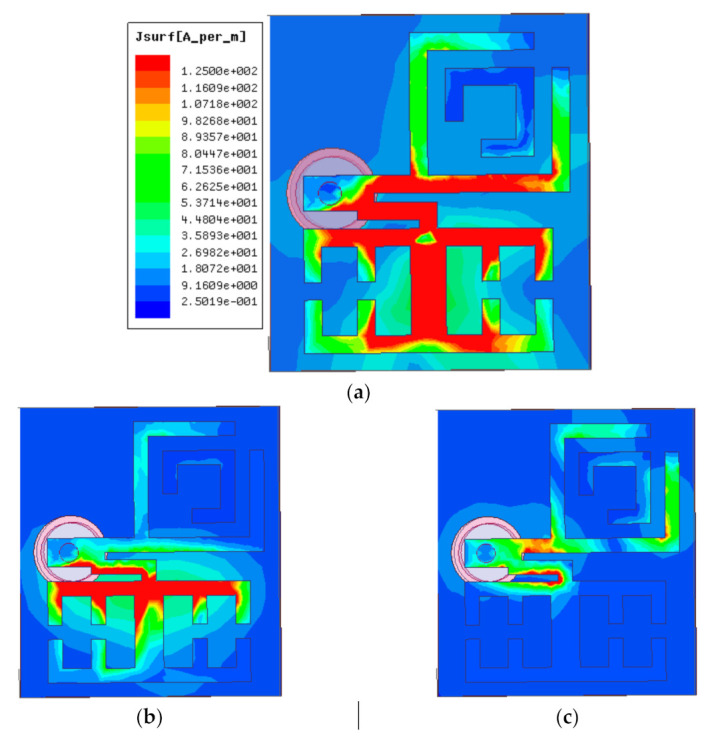
Surface current distributions at (**a**) 3.2 GHz, (**b**) 4 GHz, and (**c**) 5.9 GHz.

The current operation of this multiband antenna is illustrated in Figure 6. It can be visualized that the current distribution in the antenna resonant path varies for each of the operating modes.

## 6. Results and Discussion

**Figure 7:**
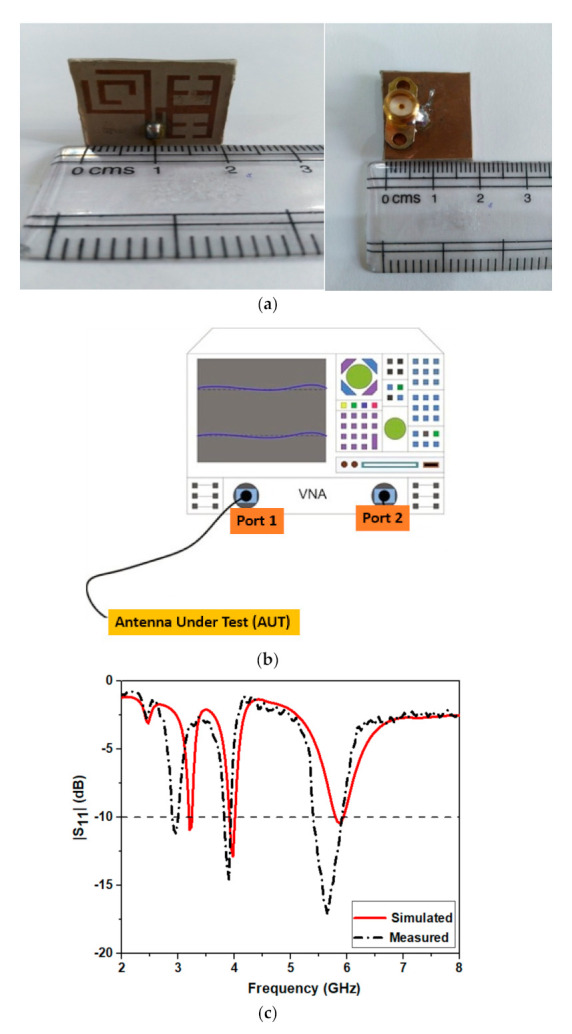
Proposed configuration (**a**) fabricated model (**b**) Schematic diagram of Vector Network Analyzer (VNA) to measure |S_11_| of the antenna and (**c**) compared simulated and measured |S_11_|.

The model of this fabricated antenna is demonstrated in Figure 7a. This model is fabricated using the photographic etching procedure. The block diagram to measure |S_11_| of the antenna from vector network analyzer is illustrated in Figure 7b. The |S_11_| of the antenna is measured using two port network analyzers, wherein at one port the antenna is kept and its readings are observed. The port 2 is terminated to a perfect matching load of 50 ohms. The compared simulated and measured |S_11_| results of the said antenna are described in Figure 7c. The antenna shows operation at 3.2 (WiMAX), 4 and 5.9 GHz under simulation with |S_11_| < −10 dB bandwidth of about 3.12%, 2.5% and 2.54%. In the measurement, the antenna shows operation at 2.9, 3.9 and 5.8 GHz with |S_11_| < −10 dB bandwidth of about 3.39%, 3.11% and 9.28%. It is observed that there is a good correlation between the measured and simulated results.

**Figure 8:**
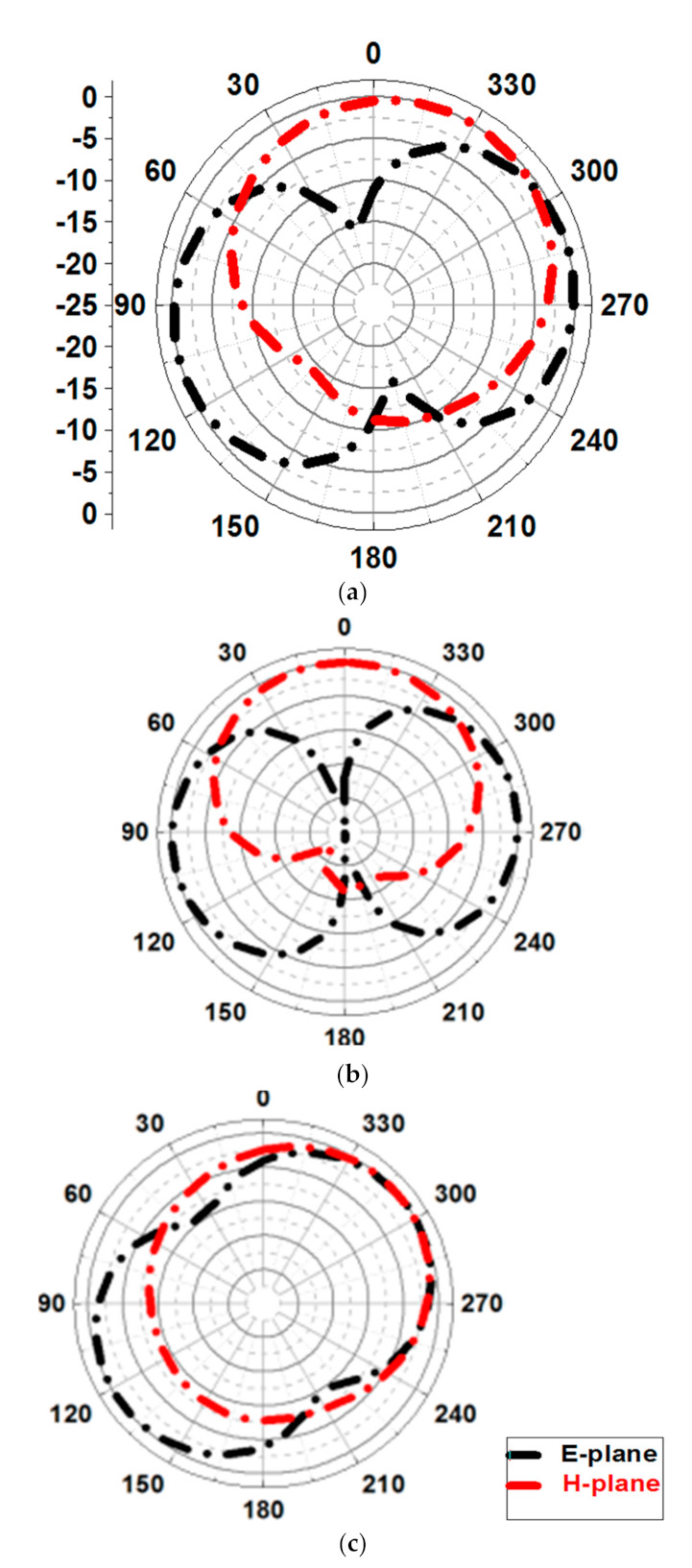
Simulated radiation patterns at (**a**) 3.2 GHz (**b**) 4 GHz (**c**) 5.9 GHz.

The simulated radiation pattern of the antenna in the E and H planes are depicted in Figure 8. It can be seen that the antenna exhibits stable radiation pattern in both the plane. Moreover, the antenna shows dipole like stable radiation patterns (i.e., H-plane omni-directional and E-plane dumbbell shaped) which is best suited for the aforementioned applications.

**Figure 9:**
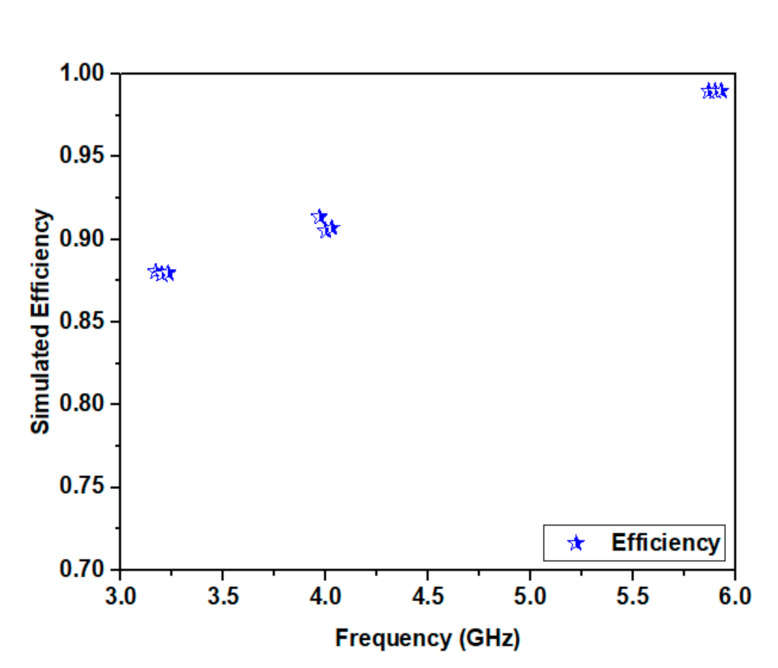
Proposed configuration simulated radiation efficiency.

The simulated radiation efficiency of the antenna is represented in Figure 9. This antenna exhibits an efficiency of 87%, 90%, and 98% at 3.2, 4.0, and 5.9 GHz, respectively.

Table 2 provides a comparative analysis of miniaturized multiband antenna proposed in the current work and with those investigated in the literature. It can be observed from Table 2 that the design proposed in this current paper gives a better insight when compared to the work in open literature in terms of size, multiband operation, and design complexity.

## 7. Conclusions

A new multiband antenna loaded with metamaterial is presented. The major advantage of this proposed design lies in its compact size and simple and planar metamaterial structure which independently controls a particular band by utilizing a particular unit cell. It is shown that the split gaps of the proposed unit cell play a significant role in determining the frequency of resonance. The proposed configuration resonates at triple band with stable radiation characteristics that make it quite attractive for WiMAX and WLAN applications.

## Figures and Tables

**Table 1:** Optimized final dimension of the proposed metamaterial antenna.

Parameters	Dimension (mm)
L	20
W	18
F	3.5
F_W_	2
M_L_	9
M_1_	5
M_S_	2
F_C_	5.1
M = M_2_ = M_3_ = S_1_ = S_2_ = S_R_	1
S_L_	14
S_W_	7
S_3_ = S_4_	2

**Table 2:** Comparative analysis of the proposed design.

Ref.	Size (mm^2^)	Operating Bands (GHz)	Metamaterial Structure	Antenna Design Technique	Metamaterial Property Verified
[15]	40 × 45	2.44/3.5/5.5	Rectangular stub	Monopole with inverted L-shaped slot as the radiating plane and partial ground plane with thin inductive stub loaded with rectangular patch	No
[17]	20 × 22	2.48/3.49	SRR	Meandered line square shaped SRR monopole fed with asymmetric CPW	No
[18]	30 × 20	5.63	CSRR	Patch loaded with two CSRR	No
[19]	30 × 40	2.4	CSRR	Rectangular patch with loaded CSRR on front and ground part	No
[20]	42 × 50	1.8/5.2	three zeroth order resonator (ZOR) cells	Rectangular patch with L-shaped ground	No
[24]	23 × 26	2.5/3.6/5.8	Triangular shaped split ring resonator	Strip line electrically coupled with metamaterial unit cell on either side	Yes
[25]	50 × 40	2.4/3.5/5.8	SRR	Two pair of symmetrical SRR with the initial monopole on the front side	Yes
[26]	40 × 12	2.5/3.5	Stepped Impedance closed ring resonator (SICRR)	Rectangular patch with partial ground plane	No
[27]	20 × 30	2.1/7.3	CRLH unit cell	Annular resonator with partial ground plane	No
[28]	28 × 21	2.4/5.2	metamaterial-based electromagnetic bandgap (MTM-EBG)	Rectangular patch with EBG at the edges	Yes
[29]	20 × 30	1.2/6.0	ZOR unit cell	Monopole antenna loaded with series inductor and capacitor	No
Prop.	20 × 18	3.2/4/5.9	CSSRR and SCSRR	Rectangular stub loaded CSRRR and SCSRR as the radiating part and full ground plane	Yes

## Data Availability

Not applicable.
